# Sex Similarities and Differences in Intelligence in Children Aged Two to Eight: Analysis of SON-R 2–8 Scores

**DOI:** 10.3390/jintelligence7020011

**Published:** 2019-05-01

**Authors:** Dorota Buczyłowska, Pola Ronniger, Jessica Melzer, Franz Petermann

**Affiliations:** Center for Clinical Psychology and Rehabilitation, University of Bremen, 28359 Bremen, Germany; ronniger@uni-bremen.de (P.R.); jmelzer@uni-bremen.de (J.M.); fpeterm@uni-bremen.de (F.P.)

**Keywords:** intelligence, sex differences, cognitive development, SON-R 2–8

## Abstract

The aim of this study was to investigate sex similarities and differences in visuospatial and fluid abilities and IQ scores based on those abilities in children aged two to eight. Standardization data from the Snijders-Oomen Nonverbal Intelligence Test for Children aged 2–8 (SON-R 2–8) were used. A representative sample composed of 965 children from the Netherlands and 762 children from Germany was examined. Small but significant mean sex differences favoring girls were observed until age four. At ages six and seven, boys achieved similar cognitive development levels to girls regarding all abilities assessed and outperformed girls on the Mosaics subtest measuring visuospatial cognition. Boys also displayed higher variability rates in performance. The distribution of IQ scores, with the overrepresentation of girls scoring above mean and the overrepresentation of boys scoring below mean in early childhood, altered with age towards parity between the sexes. The results suggest that girls tend to mature earlier with respect to cognitive abilities. During the course of development, however, the differences between girls and boys may become negligible.

## 1. Introduction

Sex differences in intelligence have been frequently researched and controversially debated. In particular, the superiority of males on visuospatial and quantitative abilities and the superiority of females on verbal abilities have been reported, both in children and adults [1,2,3,4]. As these differences are usually small or appear in a small number of tests [5,6,7], an alternative research strategy targeting at investigating and theorizing sex similarities has been proposed [8]. It has also been suggested that cognitive sex differences are rapidly changing, which may reflect changes in sociodemographic conditions [9]. For example, environmental factors such as socialization practices, gender equity, access to education, and employment may induce increases or decreases in certain cognitive sex differences. Consequently, both similarities and differences in cognition between males in females should be reexamined [9].

In particular, sex similarities and differences in cognitive development require further investigations as it is not clear at each age sex differences in cognition emerge [10]. Several studies have reported negligible or null differences in general intelligence (g) between boys and girls in primary school children and adolescents [11,12,13,14,15] or higher g scores for girls [16,17,18,19]. Fewer studies have been dedicated to IQ differences in children younger than five. Sellers et al. [20] reported no differences in g between boys and girls aged three to seven based on the standardization sample of the Wechsler Preschool and Primary Scale of Intelligence–Revised (WPPSI-R) [21]. Burns and Reynolds [22] detected an advantage for girls aged two to four on the Kaufman Assessment Battery for Children [23]. Palejwala and Fine [10] discovered a sex difference in g favoring girls aged two to seven on the Wechsler Primary and Preschool Scale of Intelligence—Fourth Edition (WPPSI-IV) [24]. Using an intelligence score composed of verbal and non-verbal tasks, Arden and Plomin [25] found greater IQ scores for girls than boys aged two to seven.

The exact age at which sex differences in specific abilities emerge is not clear either. The male advantage in visuospatial abilities has been reported to emerge at least by age five [7,14] or six [26]. Studies examining younger children suggest that the male advantage does not emerge until a minimum of age four [10,27]. In some aspects of visuospatial cognition, in particular mental rotation, evidence exists on boys outperforming girls at three months of age [28,29]. Nevertheless, a recent longitudinal study demonstrated an advantage for girls aged two, three, and four not only in verbal but also in visuospatial abilities [30]. Regarding fluid reasoning, no differences between boys and girls aged five to eight or older have been reported [14,16,26], nor in boys and girls younger than five [10,20].

Both biological and environmental factors have been proposed to explain sex differences in intelligence. Girls’ advantage in g in the early years has been explained by different rates of brain maturation between boys and girls [10,22,31]. Indeed, in a longitudinal neuroimaging study, evidence was found on girls’ earlier brain development [32] that may result in sex-specific developmental pathways. Further, sex differences may be accounted for by differential interactions of boys and girls with the environment based on cultural and learning experiences [9,22]. This might be the case especially for specific abilities [28,33,34].

When examining differences in intelligence between the sexes, besides differences in average scores, the focus of attention has been to differences in variance. Taking the variability of scores into account may help explain potential differences in mean scores and better understand the distribution of intelligence scores according to sex. Several studies have indicated a greater variability of intelligence scores for males than for females [19,35], with an overrepresentation of males in the lower and higher tail of the distribution [36,37,38]. Greater male variability in intelligence scores has been historically explained both by biological and environmental factors (for extensive review see [37]) or an interaction between the two sources of sex differences [36]. Looking from the developmental perspective, greater male variability has been demonstrated also for brain structure [39] and several different physical properties that are unrelated or indirectly related to intelligence [40]. Nevertheless, studies investigating sex differences in the variability of intelligence scores are scarce, especially in preschool children. In the study by Palejwala and Fine [10], no differences in the variability of any WPSSI-IV scores in two to seven years old children were evident. Importantly, this may be due to the method used, since other studies using a latent variable approach showed no differences in variability between boys and girls [14,17,26]. In contrast, a study by Arden and Plomin [25] that used an observed variable approach within the age range two to ten demonstrated greater boys’ variance at every age except age two, with girls being overrepresented at the high tail and boys being overrepresented at the low tail of the IQ score distribution at ages two, three, and four.

The aim of the current study was to investigate sex similarities and differences in visuospatial and fluid abilities and IQ scores solely based on those abilities in children aged two to eight. In particular, mean scores, variability in performance, and the distribution of IQ scores were analyzed. An observed variable approach based on the Snijders-Oomen Nonverbal Intelligence Test for Children aged 2–8 (SON-R 2–8, [41]) standardization data was used. A large representative sample composed of children from the Netherlands and Germany was examined. In line with the previous research, girls were expected to display higher IQ scores, whereas boys were expected to demonstrate higher variability in performance. Further, the overrepresentation of girls in the top half of the IQ distribution and the overrepresentation of boys in the bottom half of the IQ distribution were predicted.

## 2. Materials and Methods 

### 2.1. Participants

Data used in the current study were collected within the standardization of SON-R 2–8 [42]. 1727 children—877 boys and 850 girls—aged 2–8 years were tested. Data collection in the Netherlands took place in spring and summer 2016. The German part of the sample was collected from spring 2016 to spring 2017. The children were recruited and tested in day nurseries, kindergartens, and primary schools. Tests were conducted in separated rooms by psychologists, social pedagogues, and psychology students, all of whom were extensively trained in the SON-R 2–8 administration procedures. Both the Dutch and German part of the norming sample are nationally representative according to population censuses with respect to demographical characteristics such as region, degree of urbanization, migration background, and mother’s education level. No significant differences between the Dutch and German part of the sample with respect to age, sex, migration background, or mother’s and father’s education level were found [42]. The sample includes children with the following disorders: Hearing impairment (1.3%), language impairment (3%), cognitive impairment (0.5%), and autism spectrum disorder (0.5%). Sample characteristics regarding country, sex, and age are presented in Table 1.

### 2.2. Measures

The SON-R 2–8 is the fourth edition of Snijders-Oomen Nonverbal Intelligence Test for children and a revision of SON-R 2½–7 [43]. The first SON intelligence test was designed over 70 years ago in the Netherlands by Snijders-Oomen [44] and dedicated to deaf children. Subsequent SON revisions (e.g., [45]) were complemented by norms for hearing children and additional age groups. Standardized verbal and nonverbal instructions are provided in the SON-R 2–8 manual. As the test can be used without spoken language, it is particularly appropriate for children with hearing or language impairments as well as children with migration backgrounds.

The adaptive approach of test administration is used; that is, test items are presented according to the age and performance level of child. Different starting items are administered with children of the age groups 2–3, 4–5, and ≥ 6 years. In general, the administration of each subtest is terminated after three errors, even if the errors are not made in sequence. In the second part of the Puzzles, Patterns, and Mosaics subtests, however, the subtest administration is also terminated after two subsequent errors.

The SON intelligence tests are not based on any specific theory of intelligence. Nevertheless, from the first edition, the SON tests have been designed to measure visuospatial abilities, as well as abstract and concrete reasoning based on language-free tasks and nonverbal instructions. According to the Cattell-Horn-Carroll (CHC) theory [46], the SON tests may be considered measures of two broad abilities Fluid Reasoning (Gf) and Visual Processing (Gv). This is in contrast to several modern intelligence tests assessing also other components of intelligence [46].

The SON-R 2–8 consists of six subtests: Puzzles, Categories, Patterns, Situations, Mosaics, and Analogies (for details see the following section). Standard scores are provided for each subtest based on the Wechsler scale (i.e., *M* = 10, *SD* = 3). Norms are available according to age, from 2.0 years to 7.11 years, with age intervals of 1 month. Scores on all subtests are subsumed to a sum score, which is normed using the IQ scale (i.e., *M* = 100, *SD* = 15). Besides to the full IQ scale (IQ), two additional IQ scaled scores can be calculated. Scores on Puzzles, Patterns, and Mosaics are subsumed to the Performance Subscale (PS IQ) measuring spatial-perceptual, visuoconstructive, and visuomotor abilities. The PS IQ may be considered a Gv measure according to the CHC taxonomy. Nevertheless, in all three PS IQ subtests, psychomotor abilities (Gp) and psychomotor speed (Gps) are required—in particular, finger dexterity (P2) and movement time (MT). Scores on Categories, Situations, and Analogies are subsumed to the Reasoning Subscale (RS IQ) measuring abstract and concrete reasoning. Within the CHC taxonomy, the RS IQ may be considered a Gf measure.

### 2.3. SON-R 2–8 Subtests 

#### 2.3.1. PS IQ (Gv) Subtests

##### Puzzles

The subtest Puzzles consists of 14 items measuring visuomotor and visuoconstructive skills and the ability to identify spatial-perceptual relations. According to the CHC taxonomy, this subtest may measure Gv narrow abilities such as visualization (Vz), flexibility of closure (CF), and closure speed (CS). Additionally, it may measure general knowledge (K0), which is a comprehension–knowledge (Gc) narrow ability. In the first part, children are to reproduce puzzles consisting of three parts according to a template. In the second part, puzzles depicting objects of increasing complexity are to be created from three to six puzzle pieces without any template within a time limit of 120 s.

##### Patterns

The subtest Patterns consists of 16 items measuring visuomotor coordination, the ability to analyze perceptual relations, and planning abilities. In line with the CHC framework, this subtest measures Gv narrow abilities such as spatial scanning (SS) and length estimation (LE). Templates with patterns composed of dots and lines are presented. Children are to copy the patterns by connecting dots with a pencil. In the first part, all drawings are first demonstrated by the test examiner. In the second part, children are to reproduce patterns without any previous demonstration within a time limit of 120 s.

##### Mosaics

The subtest Mosaics consists of 15 items assessing the ability to analyze spatial-perceptual relations, visuomotor and visuoconstructive skills. In accordance with the CHC taxonomy, this subtest may measure Gv narrow abilities such as visualization (Vz) and length estimation (LE). In the first part, templates depicting simple figural patterns are presented. Children are to reproduce those patterns using a set of three, four, or five red squares. In the second part, patterns of increasing complexity are to be reproduced using red, yellow, and red-yellow squares within a time limit of 120 s.

#### 2.3.2. RS IQ (Gf) Subtests

##### Categories 

The subtest Categories consists of 15 items measuring the ability to identify similarities and categorize objects. According to the CHC taxonomy, this subtest may be considered a measure of the Gf narrow ability Induction (I). In the first part, children are to allocate 4–6 cards to two presented categories. In the second part, children are shown pictures of three objects that belong to the same category and are to select two out of five further objects that belong to that category.

##### Situations

The subtest Situations consists of 13 items measuring reasoning, the ability to identify perceptual relationships between people and objects, and the ability to understand complex situations. In line with the CHC taxonomy, this subtest assesses the Gf narrow ability general sequential reasoning (RG). Additionally, it may measure the Gv narrow ability CS and the Gc narrow ability K0. In the first part, four pictures are presented. The lower half or right part of each picture is missing. Children are to complete the picture by allocating the corresponding card. In the second part, pictures of different performance situations lacking one or two parts are presented. Children are to select the correct card completing the picture.

##### Analogies

The subtest Analogies consists of 17 items measuring concept formation, categorization, reasoning, and the ability to identify and apply analogies. In accordance with the CHC taxonomy this subtest may be considered a measure of the Gf narrow ability induction (I). In the first part, pictures of geometrical shapes (square, circle, triangle) varying in color, shape or size are presented. Children receive three to five pieces of plastic geometrical shapes and are to allocate them according to the presented pictures. Children are required to identify and apply the underlying rule. In the second part, presented geometric shapes change in color, shape, or size. Children are to identify the changing principle and apply it to other figures.

### 2.4. Procedure and Statistical Analysis

Statistical analyses were performed using SPSS (version 24) and Microsoft Office Excel 2007. Age-adjusted standard scores were used in all analyses. A two-way multivariate analysis of variance (MANOVA) was applied to all six subtests, and a two-way analysis of variance (ANOVA) was separately applied to the three composite scores to examine the differences in performance according to age and sex in the full sample. In order to examine the effect of sex across age more accurately, the age range two to eight was divided into six age groups: 2.0–2.11, 3.0–3.11, 4.0–4.11, 5.0–5.11, 6.0–6.11, and 7.0–7.11. Separate MANOVAs were run in all age groups with sex as independent variable and the six subtests and as dependent variables. The same analyses were separately applied to the three composite scores. Due to multiple comparisons, the Bonferroni correction was used to adjust for type I error accumulation.

Further, the data were analyzed descriptively to gain more insight into sex differences both in average performance and variability in scores as well as sex similarities and differences in the distribution of scores. Cohen’s d [47], which is the difference between means divided by the pooled within group standard deviation, was used as effect size for differences in average scores (d ≤ 0.2 = small effect size, d ≤ 0.5 = medium effect size, and d ≤ 0.8 = large effect size). To investigate the variability in the scores, the variance ratio (VR) was used. The VR is the ratio of male variance to female variance, computed by dividing the male variance by corresponding female variance. A VR greater than 1.00 implies greater variability for males than for females, a VR less than 1.00 implies greater variability in the scores for females, and a VR of 1.00 indicates homogeneity of variance across sex. VRs are considered effect sizes for differences in variability [8,47], with values between 0.90 and 1.10 indicating negligible differences between the sexes or a homogeneity of variance [36,48]. Additionally, Levene’s test of homogeneity of variance was applied to check for significant differences in variance between boys and girls.

To evaluate differences in the distribution of scores, IQ scores’ standard deviations were inspected, starting from 3 SDs below mean (i.e., IQ = 55–70) and continuing up to 3 SDs above mean (i.e., IQ = > 130–145). Additionally, chi² tests were used to check for significant differences between the number of boys and the number of girls scoring in the first, second, or third SD below and above mean.

## 3. Results

The MANOVA applied to the six subtests in the full sample yielded a significant multivariate effect of sex—Wilks’ Lambda = 0.98, *F* (6, 1710) = 6.06, *p* < 0.001, η² = 0.02—and age—Wilks’ Lambda = 0.94, *F* (30, 6842) = 3.36, *p* < 0.001, η² = 0.01. The factor of age significantly influenced the scores on Patterns—with *F* (5, 1715) = 4.29, *p* = 0.001, η² = 0.01—and Situations—with *F* (5, 1715) = 6.70, *p* < 0.001, η² = 0.02. A significant univariate effect of sex was evident for Patterns: *F* (1, 1715) = 11.73, *p* < 0.001, η² = 0.01; Categories, *F* (1, 1715) = 10.08, *p* = 0.002, η² = 0.01; and Situations, *F* (1, 1715) = 10.28, *p* = 0.001, η² = 0.01.

A multivariate effect of interaction between age and sex was significant as well—Wilks’ Lambda = 0.98, *F* (30, 6842) = 1.46, *p* = 0.049, η² = 0.01—with a significant univariate effect for Mosaics, *F* (5, 1715) = 4.33, *p* = 0.001, η² = 0.01.

The ANOVAs applied to the three composite scores in the full sample yielded a significant effect of sex for IQ—*F* (1, 1715) = 6.82, *p* = 0.009, η² = 0.01; and IQ RS, *F* (1, 1715) = 12.26, *p* < 0.001, η² = 0.01. While the effect of age was not significant in any of the three analyses, the interaction between age and sex was significant for IQ—*F* (5, 1715) = 2.34, *p* = 0.040, η² = 0.01.

Subsequent MANOVAs applied to the six subtests (see Table 2) and ANOVAs applied to the three composite scores (see Table 3) conducted in the individual age groups provide better insight into the effect of sex and the interaction between sex and age. In the two-year age group, a multivariate effect of sex was significant—Wilks’ Lambda = 0.95, *F* (6, 263) = 2.57, *p* = 0.020, η² = 0.06. Girls outperformed boys on Patterns and Situations.

A multivariate effect of sex was not significant at ages three, four, five, and six. Nevertheless, at age four, a multivariate effect of sex slightly missed the significance level—Wilks’ Lambda = 0.96, *F* (6, 284) = 2.11, *p* = 0.053, η² = 0.04—and a univariate effect of sex was significant for Categories with girls outperforming boys. At age six, both a multivariate effect of sex—Wilks’ Lambda = 0.96, *F* (6, 283) = 2.10, *p* = 0.054, η² = 0.04—and a univariate effect of sex for Mosaics (boys outperforming girls) slightly missed the significance level. A multivariate effect of sex was significant in the seven-year age group—Wilks’ Lambda = 0.94, *F* (6, 285) = 2.81, *p* < 0.011, η² = 0.06—with a significant univariate effect of sex for the Mosaics subtest with boys outperforming girls.

As presented in Table 3, the girls outperformed the boys in the two-year age group on all three IQ scores and in the four-year age group on IQ RS.

The standardized mean differences in subtest and IQ scores were rather small, even in the two-year olds, with d values of −0.34, −0.36, and −0.40, respectively, for IQ PS, IQ RS, and IQ. The magnitude of d values also decreased with age.

As presented in Table 2 and Table 3, the descriptive analysis revealed meaningful differences in the variability of scores between the sexes already in early childhood. That is, in the two-year age group, the VRs indicated greater boys’ variance in Mosaics, Categories, IQ PS, and IQ. No differences were found at age three. The boys had also greater variance than girls in the four-year age group in Patterns, Mosaics, and all IQ scores; in the five-year age group in Patterns, Mosaics, Situations, and all IQ scores; in the six-year age group in Mosaics, Categories, IQ RS, and IQ; and in the seven-year age group in Mosaics and IQ. Nevertheless, Levene’s tests of homogeneity of variance revealed significantly greater variance for boys than for girls only in the four-year age group for Patterns: *F* (1, 289) = 8.14, *p* = 0.005 and Mosaics, *F* (1, 289) = 5.94, *p* = 0.015; in the five-year age group for Mosaics: *F* (1, 291) = 14.39, *p* < 0.001, IQ RS, *F* (1, 291) = 4.60, *p* = 0.033, and IQ, *F* (1, 291) = 5.70, *p* = 0.018; and in the six-year age group for Categories: *F* (1, 288) = 5.23, *p* = 0.023.

**Figure 1:**
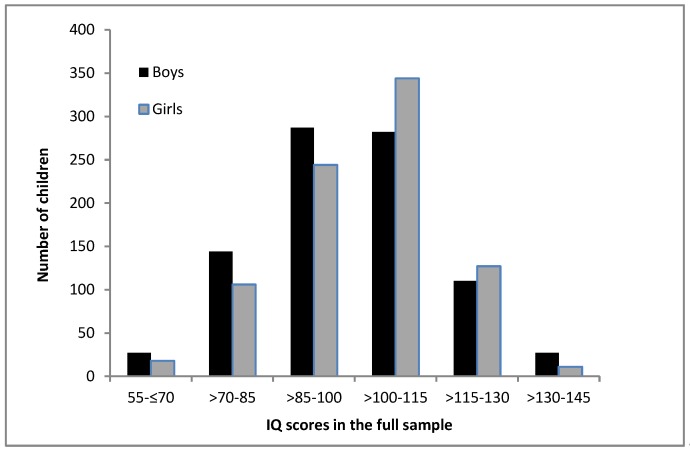
Distribution of IQ scores for boys and girls in the full sample.

**Figure 2:**
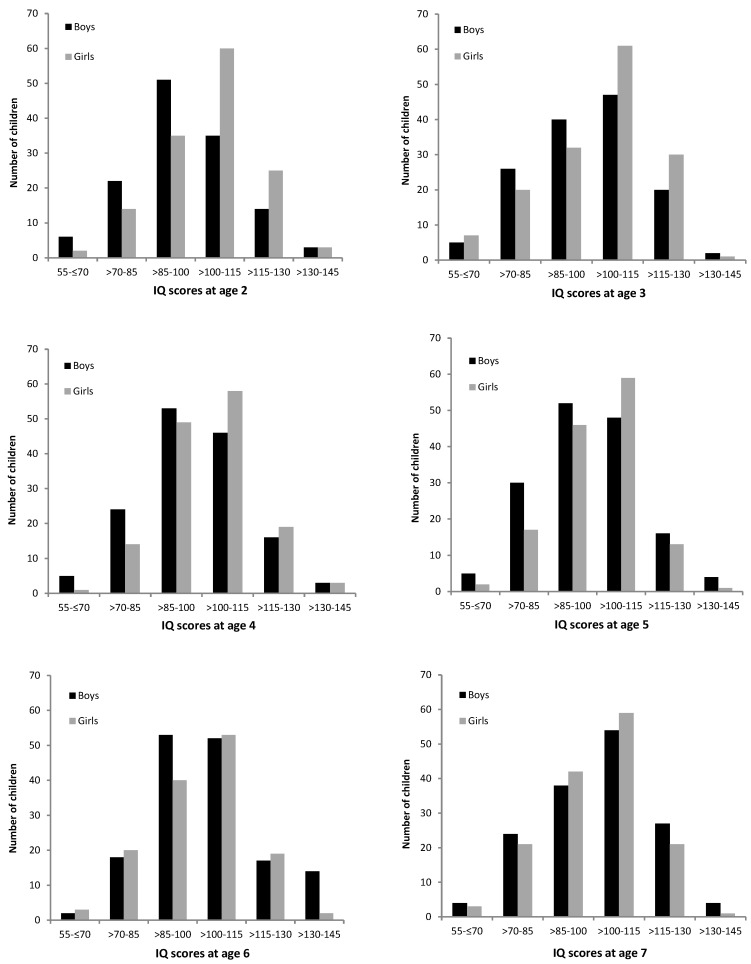
Distribution of IQ scores for boys and girls according to age group.

The inspection of the distribution of IQ scores in the full sample (see Figure 1) revealed that the boys were overrepresented in the bottom half of the IQ distribution, with significant differences between the sexes in the second SD below mean—χ² (1) = 5.78, *p* = 0.016. The girls were overrepresented in the top half of the IQ distribution. However, although there were significantly more girls in the first SD above mean—χ² (1) = 6.14, *p* = 0.013—the boys were overrepresented in the third SD above mean—χ² (1) = 6.74, *p* = 0.009. When inspecting the distribution of IQ scores according to age group, as depicted in Figure 2, it appears that the differences in the distribution of IQ scores between the boys and girls decreased with advancing age. Indeed, in the two-year old age group, chi² tests revealed significant differences between the boys and girls in the IQ scores distribution, with the overrepresentation of girls in the first SD above mean—χ² (1) = 6.58, *p* = 0.010. In contrast, in other age groups no significant differences between the boys and girls were detected, with the exception of six-year old children, where the boys were overrepresented in the third SD above mean—χ² (1) = 6.23, *p* = 0.013.

## 4. Discussion

The results imply that the girls tend to display better performance on all SON-R 2–8 subtests when considering two-year-old children, with significant differences in Patterns and Situations subtests and all three intelligence scores. The girls also outperform the boys at age four on the Categories subtest and RS IQ. Nevertheless, as suggested by the significant effect of interaction between age and sex for Mosaics and IQ, a shift in performance between the girls and boys can be observed in six- and seven-year old children. Though the boys showed significantly better performance than the girls only on the Mosaics subtest, the descriptive statistics suggests that at age six and seven, the boys achieve a similar development level to the girls on all SON-R 2–8 subtests. Moreover, in contrast to age four, at age five no significant differences between the sexes were found. Thus, the current research suggests that differences in cognitive performance favoring girls exist up to age four. As reviewed in the introduction, the majority of previous studies targeting similar age ranges [10,22,25] demonstrated higher IQ scores for girls up to age seven. The current study, however, examined intelligence using tasks measuring solely Gv and Gf, whereas the previous studies measured a broader spectrum of cognitive abilities including verbal skills and processing speed. This seems important, as IQ scores are typically calculated on the basis of different tasks measuring specific abilities. As an example, better girls’ performance on processing speed has been demonstrated in children four to seven [10]. As processing speed scores often contribute to IQ overall scores, the girls’ advantage on processing speed may result in girls’ higher IQ scores. Consequently, when investigating sex similarities and differences in cognitive development, the nature of tasks contributing to IQ scores must be considered. Accordingly, given the composition of the IQ overall scores analyzed in the current study, it must be emphasized that they are not measures of general intelligence.

With respect to Gv, the current study supports the theory that the male advantage may not emerge in early childhood [10,27], as a significant male advantage was evident only for the Mosaics subtest and emerged at age seven. Moreover, girls outperformed the boys on Patterns at age two. Consequently, the current results contribute to the previous evidence demonstrating an advantage in some aspects of visuospatial cognition for girls aged two to four [30]—spatial scanning and length estimation within the CHC taxonomy, in particular.

In contrast to previous studies investigating Gf in preschoolers [10,20], the current findings suggest a slight female advantage, since the girls outperformed the boys on the Situations subtest in two-year old children, on the RS subtest Categories, and on RS IQ in the four-year old children. The Categories subtest as a measure of the CHC narrow ability Induction requires the ability to identify similarities and categorize objects that is also considered part of executive functioning. Thus, in early childhood, girls may also display an advantage in some aspects of executive performance.

In line with the previous research [10,22], standardized mean differences both for subtests and IQ scores were rather small with the magnitude of d values decreasing with advancing age. This suggests that girls tend to mature earlier with respect to cognitive abilities. The differences between girls and boys, however, become negligible during the course of development.

In the current study, differences in the variability of scores between the boys and girls differed according to subtest or composite score and were more pronounced for IQ scores as well as Gv tasks. As expected, in all cases the boys displayed greater variance in performance than the girls; in particular, boys’ performance on the Mosaics subtest, measuring visualization, and length estimation was more variable for the boys in all age groups with the exception of the three-year olds. This is consistent with the previous research that showed greater male variability on visuospatial abilities [49,50].

Nevertheless, differences in variance were significant only in the four, five, and six-year-old children and evident only for a few scores. Consequently, although there is a trend for greater boys’ variability starting already at age two, the differences between the sexes are rather small and slightly decrease with advancing age. Moreover, at age seven, the variance differences become similar in their magnitude for all abilities assessed.

Consistent with the previous findings [19,25,37,38], the current research demonstrated greater boys’ variance and the overrepresentation of boys in the lower and higher tail of the distribution when examining the full age range two to eight. Nevertheless, the differences in the IQ score distribution were less pronounced than in the previous studies and less consistent across age. This may be due to the sample size, as the current study’s sample might be rather small to detect differences in the distribution of scores. The previous studies were often much larger and thus more accurate in drawing interferences on the distribution of intelligence in the population. In the current study, the girls were significantly overrepresented in the first SD above mean, both in the full sample and in the two-year age group, and the boys were overrepresented in the second SD below mean in the full sample and in the third SD above mean at age six. Furthermore, the differences in the distribution of IQ scores between the boys and girls decreased with advancing age. Consequently, these findings may be explained by sex-specific developmental pathways related to differential trajectories of brain development for girls and boys [10,22,31]. Due to the girls’ accelerated brain development [32], girls may achieve higher average scores in the early years and be overrepresented in the top half of the IQ distribution. Boys, however, are behind girls in terms of brain maturation and show lower average scores and higher variance and, thus, might be overrepresented in the bottom half of the distribution. With advancing age, nevertheless, boys appear to display similar development levels or start to outperform girls in some Gv abilities, such as visualization and length estimation. As a result, when considering intelligence measures considerably based on Gv abilities, boys might be overrepresented among high achievers.

## 5. Conclusions

Based on SON-R 2–8 scores within the age range four to eight, mean sex differences in Gv and Gf favoring girls may exist until age four and are rather small. At ages six and seven, boys achieve similar development levels in intelligence to girls and start to outperform girls on some aspects of Gv abilities—visualization and length estimation, in particular. Boys appear to be more variable in performance, but, as with the magnitude of average mean differences, the magnitude of differences in variability between the sexes may decrease with advancing age. The distribution of IQ scores with the overrepresentation of girls scoring above mean and the overrepresentation of boys scoring below mean in early childhood alters with age towards parity between the sexes. The current results imply that girls tend to mature earlier with respect to cognitive abilities. During the course of development, however, the differences between girls and boys may become negligible. Especially within the assessment of cognitive abilities and therapy of cognitive impairments, the differential developmental pathways for boys as girls that might exist in the early childhood should be considered. Further, as boys start to excel or outperform girls at some aspects of visuospatial cognition at age seven, potential sources of these differences, especially those related to cultural and learning experiences, should be better explored. 

## 6. Limitations and Recommendations for Future Research

The current study is based on cross-sectional data; thus, all conclusions are constrained by the limitations usually related to cross-sectional study design [51]. In future studies, a longitudinal study design investigating the same participants at different ages will be required. Further, the current study is based on a norming sample representative for the population and thus appears appropriate to examine differences and similarities in cognitive abilities. Nevertheless, to draw conclusions about the distribution of IQ scores, larger samples composed of several thousand participants are recommended. The conclusions from the current study might also be limited by the assessment tool used. As the IQ scores analyzed were substantially composed of Gv and Gf abilities, interferences only with respect to those abilities can be drawn. Future research should use tasks measuring different aspects of cognition as well [52,53], with a particular emphasis on Gs and Gc abilities.

## Figures and Tables

**Table 1:** Sample characteristics.

	Dutch	German	Full Sample	Sex		Age
Age Group	N	N	N	Boys	Girls	Mean (SD)
2 years	144	126	270	131	139	2.52 (0.36)
3 years	165	126	291	140	151	3.50 (0.28)
4 years	165	126	291	147	144	4.50 (0.29)
5 years	165	128	293	155	138	5.52 (0.29)
6 years	162	128	290	153	137	6.48 (0.30)
7 years	164	128	292	151	141	7.48 (0.27)
Overall	965	762	1727	877	850	5.03 (1.71)

**Table 2:** Results of the MANOVAs for the SON-R 2–8 subtests including effect sizes for sex differences in mean (d) and variability (VR).

		Boys		Girls							
Age	Variable	M	SD	M	SD	Δ	F	p	p corr.	d	VR
**2 years**	Puzzles	9.82	2.86	10.09	2.69	−0.27	0.64	0.426	2.556	−0.10	1.07
	Patterns	**10.08**	**3.44**	**11.53**	**3.26**	**−1.45**	**12.64**	**0.000**	**0.000**	**−0.43**	1.06
	Mosaics	9.70	3.31	10.35	2.94	−0.64	2.85	0.093	0.558	−0.21	**1.13**
	Categories	9.79	3.18	10.51	2.80	−0.72	3.87	0.050	0.300	−0.24	**1.14**
	Situations	**8.79**	**3.55**	**10.09**	**3.49**	**−1.30**	**9.19**	**0.003**	**0.018**	**−0.37**	1.02
	Analogies	9.61	3.39	10.43	3.41	−0.82	3.92	0.049	0.294	−0.24	0.99
**3 years**	Puzzles	10.02	3.17	10.05	3.01	−0.02	0.00	0.945	5.670	−0.01	1.05
	Patterns	10.44	3.16	10.96	3.15	−0.52	1.95	0.163	0.978	−0.17	1.00
	Mosaics	9.81	3.50	10.07	3.25	−0.25	0.41	0.525	3.150	−0.08	1.08
	Categories	9.74	3.09	10.06	2.92	−0.32	0.81	0.370	2.220	−0.11	1.06
	Situations	9.43	2.86	10.01	2.96	−0.58	2.87	0.092	0.552	−0.20	0.97
	Analogies	10.14	3.00	10.09	2.94	0.04	0.02	0.902	5.412	−0.02	1.02
**4 years**	Puzzles	9.99	2.87	10.09	2.90	−0.10	0.08	0.774	4.644	−0.04	0.99
	Patterns	10.05	2.95	10.42	2.08	−0.37	1.52	0.219	1.314	−0.15	**1.42**
	Mosaics	9.90	2.90	10.54	2.39	−0.64	4.17	0.042	0.252	−0.24	**1.22**
	Categories	**9.53**	**3.18**	**10.62**	**3.05**	**−1.09**	**8.86**	**0.003**	**0.018**	**−0.35**	1.04
	Situations	9.59	2.86	10.05	2.68	−0.46	1.97	0.161	0.966	−0.17	1.07
	Analogies	9.65	2.82	10.16	2.80	−0.51	2.37	0.125	0.750	−0.18	1.01
**5 years**	Puzzles	9.91	3.01	10.09	3.00	−0.18	0.28	0.600	3.600	−0.06	1.00
	Patterns	9.46	3.07	10.02	2.63	−0.56	2.82	0.094	0.564	−0.20	**1.17**
	Mosaics	10.08	3.44	9.72	2.53	0.36	1.02	0.313	1.878	0.12	**1.36**
	Categories	9.72	3.16	10.09	2.90	−0.37	1.09	0.298	1.788	−0.12	1.09
	Situations	9.87	3.10	10.37	2.66	−0.50	2.16	0.143	0.858	−0.17	**1.17**
	Analogies	9.72	3.13	10.20	2.88	−0.47	1.80	0.181	1.086	−0.16	1.08
**6 years**	Puzzles	10.07	3.34	10.09	3.13	−0.02	0.00	0.952	5.712	−0.01	1.07
	Patterns	10.29	3.35	10.30	3.16	−0.01	0.00	0.976	5.856	−0.01	1.06
	Mosaics	10.50	3.15	9.58	2.79	0.92	6.86	0.009	0.054	0.31	**1.13**
	Categories	10.23	3.42	10.19	2.84	0.04	0.01	0.916	5.496	0.01	**1.20**
	Situations	10.50	3.05	10.31	3.06	0.19	0.28	0.599	3.594	0.06	1.00
	Analogies	10.46	3.21	10.15	3.18	0.30	0.66	0.419	2.514	0.10	1.01
**7 years**	Puzzles	10.07	3.24	10.00	3.21	0.07	0.04	0.847	5.082	0.02	1.01
	Patterns	10.15	3.25	10.27	3.03	−0.12	0.11	0.737	4.422	−0.04	1.07
	Mosaics	**10.55**	**3.20**	**9.52**	**2.91**	**1.03**	**8.16**	**0.005**	**0.030**	**0.34**	**1.10**
	Categories	9.75	3.09	10.10	2.84	−0.34	0.98	0.324	1.944	−0.12	1.09
	Situations	10.62	3.07	10.78	2.92	−0.16	0.22	0.641	3.846	−0.05	1.05
	Analogies	9.87	2.88	9.88	2.65	−0.01	0.00	0.987	5.922	−0.01	1.09

Note: Significant MANOVA results and meaningful variance ratio (VR) effects (i.e., ≤0.90 and ≥1.10) are in bold.

**Table 3:** Results of the ANOVAs for the SON-R 2–8 composite scores including effect sizes for sex differences in mean (d) and variability (VR).

		Boys		Girls							
Age	Variable	M	SD	M	SD	Δ	F	p	p corr.	d	VR
**2 years**	IQ PS	**99.09**	**15.50**	**104.06**	**13.89**	**−4.97**	7.70	**0.006**	**0.018**	**−0.34**	**1.12**
	IQ RS	**96.32**	**16.19**	**102.09**	**15.99**	**−5.77**	8.68	**0.003**	**0.009**	**−0.36**	1.01
	IQ	**97.44**	**15.55**	**103.37**	**14.20**	**−5.93**	10.73	**0.001**	**0.003**	**−0.40**	**1.10**
**3 years**	IQ PS	100.51	16.39	102.15	16.11	−1.64	0.74	0.391	1.173	−0.10	1.02
	IQ RS	98.24	15.84	100.44	15.18	−2.19	1.46	0.229	0.687	−0.14	1.04
	IQ	99.57	16.07	101.52	15.59	−1.95	1.11	0.294	0.882	−0.12	1.03
**4 years**	IQ PS	100.01	14.95	102.02	12.25	−2.01	1.57	0.212	0.636	−0.15	**1.22**
	IQ RS	**97.22**	**16.34**	**101.89**	**14.48**	**−4.67**	6.65	**0.010**	**0.030**	**−0.30**	**1.13**
	IQ	98.50	15.25	102.17	13.01	−3.67	4.87	0.028	0.084	−0.26	**1.17**
**5 years**	IQ PS	98.99	15.54	99.58	13.16	−0.59	0.12	0.729	2.187	−0.04	**1.18**
	IQ RS	98.19	16.49	101.35	13.60	−3.16	3.16	0.077	0.231	−0.21	**1.21**
	IQ	98.46	15.75	100.38	12.91	−1.92	1.28	0.258	0.774	−0.13	**1.22**
**6 years**	IQ PS	101.76	16.17	100.18	15.07	1.58	0.74	0.391	1.173	0.10	1.07
	IQ RS	102.76	16.93	101.66	15.04	1.10	0.34	0.561	1.683	0.07	**1.13**
	IQ	102.41	16.05	100.79	14.59	1.62	0.80	0.372	1.116	0.11	**1.10**
**7 years**	IQ PS	101.41	16.09	99.44	15.08	1.97	1.16	0.282	0.846	0.13	1.07
	IQ RS	100.55	16.00	101.65	14.65	−1.10	0.38	0.540	1.620	−0.07	1.09
	IQ	101.16	16.03	100.65	14.60	0.51	0.08	0.778	2.334	0.03	**1.10**

Note: Significant ANOVA results and meaningful variance ratio (VR) effects (i.e., ≤0.90 and ≥1.10) are in bold.
